# Transcriptomic and Proteomic Analysis Reveals the Potential Role of RBMS1 in Adipogenesis and Adipocyte Metabolism

**DOI:** 10.3390/ijms241411300

**Published:** 2023-07-11

**Authors:** Ghida Dairi, Saeed Al Mahri, Hicham Benabdelkamel, Assim A. Alfadda, Abdulrahman A. Alswaji, Mamoon Rashid, Shuja Shafi Malik, Jahangir Iqbal, Rizwan Ali, Maria Al Ibrahim, Khalid Al-Regaiey, Sameer Mohammad

**Affiliations:** 1 Physiology Department, College of Medicine, King Saud University, Riyadh 11362, Saudi Arabia; gsdairi@gmail.com (G.D.); kalfregaiey@ksu.edu.sa (K.A.-R.); 2 Deanship of Scientific Research, Umm Al-Qura University, Makkah 21961, Saudi Arabia; 3 Experimental Medicine, King Abdullah International Medical Research Center (KAIMRC), King Saud Bin Abdulaziz University for Health Sciences (KSAU-HS), Ministry of National Guard Health Affairs (NGHA), Riyadh 11426, Saudi Arabia; almahrisa@ngha.med.sa (S.A.M.); maliksh@ngha.med.sa (S.S.M.); alibrahimmaria1@gmail.com (M.A.I.); 4 Proteomics Resource Unit, Obesity Research Center, College of Medicine, King Saud University, P.O. Box 2925 (98), Riyadh 11461, Saudi Arabia; hbenabdelkamel@ksu.edu.sa (H.B.); aalfadda@ksu.edu.sa (A.A.A.); 5 Department of Medicine, College of Medicine, King Saud University, P.O. Box 2925 (38), Riyadh 11461, Saudi Arabia; 6 Infectious Disease Department, King Abdullah International Medical Research Center (KAIMRC), King Saud Bin Abdulaziz University for Health Sciences (KSAU-HS), Ministry of National Guard Health Affairs (NGHA), Riyadh 11426, Saudi Arabia; alswajiab@ngha.med.sa; 7 Department of AI and Bioinformatics, King Abdullah International Medical Research Center (KAIMRC), King Saud Bin Abdulaziz University for Health Sciences (KSAU-HS), Ministry of National Guard Health Affairs (NGHA), Riyadh 11426, Saudi Arabia; rashidma@ngha.med.sa; 8 King Abdullah International Medical Research Center (KAIMRC), King Saud Bin Abdulaziz University for Health Sciences, King Abdulaziz Medical City Hospital, Ministry of National Guard Health Affairs, Al Hasa 31982, Saudi Arabia; jahangir.iqbal@downstate.edu; 9 Medical Research Core Facility and Platforms, King Abdullah International Medical Research Center (KAIMRC), King Saud Bin Abdulaziz University for Health Sciences (KSAU-HS), MNGHA, Riyadh 11426, Saudi Arabia; aliri@ngha.med.sa

**Keywords:** adipogenesis, RBMS1, RNA-binding proteins, lipid metabolism

## Abstract

Adipocytes play a critical role in maintaining a healthy systemic metabolism by storing and releasing energy in the form of fat and helping to regulate glucose and lipid levels in the body. Adipogenesis is the process through which pre-adipocytes are differentiated into mature adipocytes. It is a complex process involving various transcription factors and signaling pathways. The dysregulation of adipogenesis has been implicated in the development of obesity and metabolic disorders. Therefore, understanding the mechanisms that regulate adipogenesis and the factors that contribute to its dysregulation may provide insights into the prevention and treatment of these conditions. RNA-binding motif single-stranded interacting protein 1 (RBMS1) is a protein that binds to RNA and plays a critical role in various cellular processes such as alternative splicing, mRNA stability, and translation. RBMS1 polymorphism has been shown to be associated with obesity and type 2 diabetes, but the role of RBMS1 in adipose metabolism and adipogenesis is not known. We show that RBMS1 is highly expressed during the early phase of the differentiation of the murine adipocyte cell line 3T3-L1 and is significantly upregulated in the adipose tissue depots and adipocytes of high-fat-fed mice, implying a possible role in adipogenesis and adipose metabolism. Knockdown of RBMS1 in pre-adipocytes impacted the differentiation process and reduced the expression of some of the key adipogenic markers. Transcriptomic and proteomic analysis indicated that RBMS1 depletion affected the expression of several genes involved in major metabolic processes, including carbohydrate and lipid metabolism. Our findings imply that RBMS1 plays an important role in adipocyte metabolism and may offer novel therapeutic opportunity for metabolic disorders such as obesity and type 2 diabetes.

## 1. Introduction

Obesity is a complex disease characterized by an excessive amount of body fat [1,2]. It is a major public health concern worldwide and is associated with numerous health problems such as heart disease, diabetes, and certain types of cancer [3,4,5,6,7,8,9,10]. Furthermore, obese people are more likely to develop severe disease, require hospitalization, and die as a result of viral infections, including the recent SARS-CoV-2 pandemic [11,12,13]. The key factor in the pathophysiology of obesity-related metabolic abnormalities is obesity-induced adipose tissue dysfunction [14,15]. Adipose tissue dysfunction leads to the release of pro-inflammatory cytokines and adipokines, which contribute to insulin resistance and chronic low-grade inflammation [13,14,15]. Adipose tissue is a kind of connective tissue that stores energy in the form of fat and plays a crucial role in regulating metabolism, hormone production, and immune function [16]. In obesity, adipose tissue becomes inflamed and dysfunctional, leading to insulin resistance and other metabolic disturbances that contribute to the development of chronic diseases [17]. Adipocytes, the primary component of adipose tissue, are derived from multipotent mesenchymal stem cells (MSCs) through adipogenesis, a complicated and highly regulated process. Understanding the mechanisms that regulate adipogenesis and the factors that contribute to its dysregulation may provide insights into the prevention and treatment of these conditions [18,19].

The transcriptional regulation of adipogenesis has been comprehensively understood, and several transcription factors have been identified as key regulators of this process, including peroxisome proliferator-activated receptor gamma (PPARγ), CCAAT/enhancer-binding protein alpha (C/EBPα), and sterol regulatory element-binding protein 1c (SREBP-1c) [19,20]. Recent studies have highlighted the post-transcriptional regulation of adipogenesis, including the role of microRNAs and RNA-binding proteins (RBPs) in modulating adipocyte differentiation and function [21,22,23,24]. RBPs such as HuR, QK, and YBX1 have been shown to regulate adipogenesis by controlling the stability and translation of key mRNAs involved in this process, suggesting a new avenue for therapeutic intervention [25,26,27].

RNA-binding motif single-stranded interacting protein 1 (RBMS1) is a member of the RNA-binding protein family and plays key roles in the post-transcriptional regulation of gene expression. RBMS1 has been shown to be involved in various cellular processes, including mRNA splicing and stability, as well as translation initiation. Multiple studies have shown that RBMS1 expression is altered in several types of cancer, and its dysregulation has been associated with tumor progression and metastasis [28,29,30,31]. RBMS1 may play a role in the development of coronary heart disease, possibly through its involvement in lipid metabolism and inflammation [32]. In addition, RBMS1 polymorphism has been found to be significantly associated with an increased risk of developing type 2 diabetes in several populations, suggesting a possible role in metabolic regulation as well [33,34,35]. No previous study has explored the role of RBMS1 in adipogenesis and adipose metabolism. This study was undertaken to investigate the role of RBMS1 in adipocyte differentiation and lipid metabolism. We show that RBMS1 may play an important role in the regulation of adipocyte differentiation and metabolism and may offer a novel therapeutic opportunity for metabolic disorders such as obesity and type 2 diabetes.

## 2. Results

### 2.1. RBMS1 Expression Is Upregulated in Adipose Tissue Depots and Adipocytes of Mice Fed a High-Fat Diet

**Figure 1:**
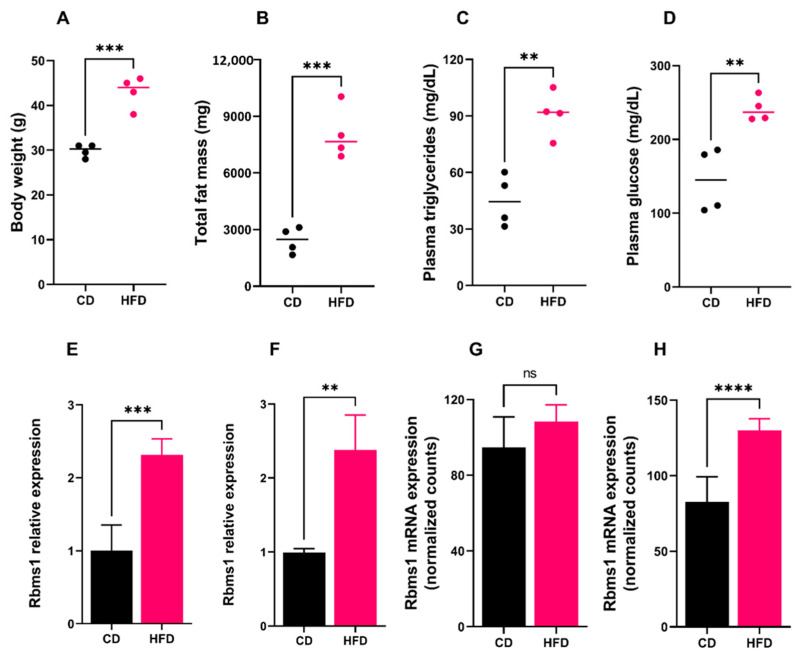
*Rbms1* expression is significantly altered in adipose tissue depots and adipocytes of mice fed a high-fat diet (HFD). (**A**–**D**) Body weight, fat mass, plasma triglyceride levels, and plasma glucose levels in the two groups of mice. (**E**,**F**) *Rbms1* expression in subcutaneous (**E**) and mesenteric adipose tissue (**F**) of mice fed either a CD or HFD. Each bar represents data from four mice from each group. (**G**,**H**) *Rbms1* mRNA expression in adipocyte fraction of visceral adipose of mice fed a CD or HFD for 8 weeks and 20 weeks. The data were obtained through re-analysis of a dataset (GSE 142187), as described in Section 4. Dots represent each value and bars represent mean ± standard deviation. RT-PCR (n = 4 in each group) and RNA sequencing (n = 6 for each group). ^ns^ *p* > 0.05, ** *p* < 0.01, *** *p* < 0.001 and **** *p* < 0.0001.

To understand the functional significance of RBMS1 in adipose tissue metabolism, we first evaluated the effect of high-fat diet (HFD) on the expression of *Rbms1* in mouse adipose tissue depots. C57BL/6J male mice (four in each group) were fed either a regular chow diet (CD) or HFD for 18 weeks, as described in Section 4. After 18 weeks, HFD-fed mice gained more weight and fat mass compared to those fed a regular diet (Figure 1A,B). Moreover, HFD mice developed hyperglycemia and hypertriglyceridemia, showing diet-induced obesity and insulin resistance (Figure 1C,D). The expression of *Rbms1* in adipose tissue depots in mice from the CD and HFD groups was subsequently assessed using quantitative real-time PCR using specific primers. Rbms1 expression increased significantly in the subcutaneous (scWAT) and visceral (vsWAT) white adipose tissue depots of HFD mice compared to CD animals (Figure 1E,F). Since diet-induced obesity (DIO) is known to alter the cellular composition of adipose tissue, which may have a direct impact on the expression of genes, including *Rbms1*, we re-analyzed an RNA sequencing dataset (GSE #142187) to evaluate the expression of *Rbms1* in the adipocyte fraction [36]. The dataset comprised RNA expression data of adipocytes extracted from white adipose tissue of mice fed a CD or HFD for 8 and 20 weeks. The mRNA expression of *Rbms1* was significantly increased in the adipocytes of HFD mice as compared to the CD mice, which is consistent with our data obtained from whole adipose tissue (Figure 1G,H).

### 2.2. RBMS1 Expression Is Downregulated during the Adipogenic Differentiation of 3T3-L1 Cells

**Figure 2:**
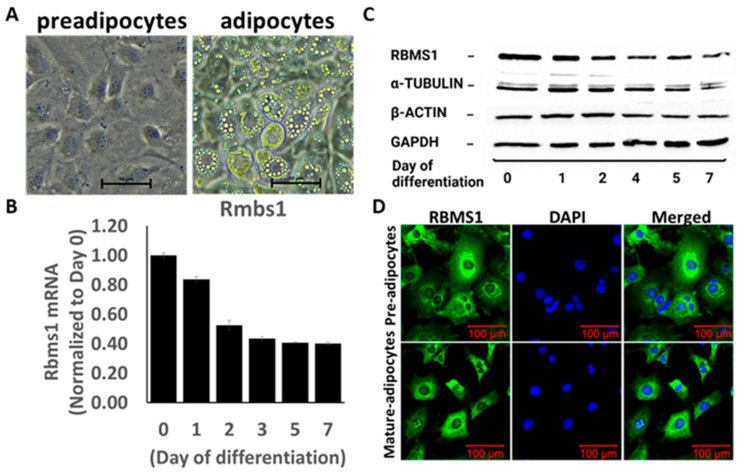
RBMS1 expression before and during adipogenic differentiation in 3T3-L1 cells. (**A**) Representative images showing 3T3-L1 cells before (pre-adipocytes) and after differentiation (adipocytes). (**B**) *Rbms1* mRNA expression during the differentiation process. Each bar represents an average of three independent experimental results; data are normalized to *Rbms1* expression on day 0. (**C**) Representative blots showing the protein expression of RBMS1 and three endogenous controls during the differentiation process. (**D**) Representative confocal images showing cellular distribution of RBMS1 in 3T3-L1 cells before and after differentiation.

3T3-L1 cells are derived from mouse embryos and are commonly used as a model for studying adipocyte differentiation and lipid metabolism. The 3T3-L1 cell line provides a useful tool for studying the molecular mechanisms underlying adipogenesis and for testing potential anti-obesity drugs. We subjected 3T3L1 cells (day 0) to RNA sequencing to assess the expression of the whole repertoire of RBPs in 3T3-L1 cells prior to differentiation (pre-adipocytes), and Rbms1 was one of the highly expressed RBPs (Supplementary Table S1). We induced the adipogenic differentiation of these cells by incubating them with differentiation cocktail of IBMX, dexamethasone, insulin, and rosiglitazone, followed by post-differentiation with insulin alone. The cells were imaged on day 7 after differentiation to confirm the differentiation process (Figure 2A). Next, we investigated how adipogenic differentiation affects RBMS1 expression by measuring RNA and protein expression at different points during the differentiation process. Interestingly, RBMS1 was highly expressed before and during the early stages of differentiation, but declined toward the end of the differentiation process (Figure 2B,C). For reliability and uniformity, we used multiple controls (β-actin, GAPDH, and α-tubulin) to assess RBMS1 protein expression at different stages of differentiation (Figure 2C). We examined the subcellular localization of RBMS1 in these cells and discovered that the protein was excluded from the nucleus and predominantly expressed in the cytoplasm, consistent with its role in RNA metabolism (Figure 2D). These findings suggest that RBMS1 may play a role in regulating adipogenesis and adipose metabolism by controlling RNA processing and transport within the cytoplasm of these cells.

### 2.3. RBMS1 May Play an Important Role in Adipocyte Metabolism

**Figure 3:**
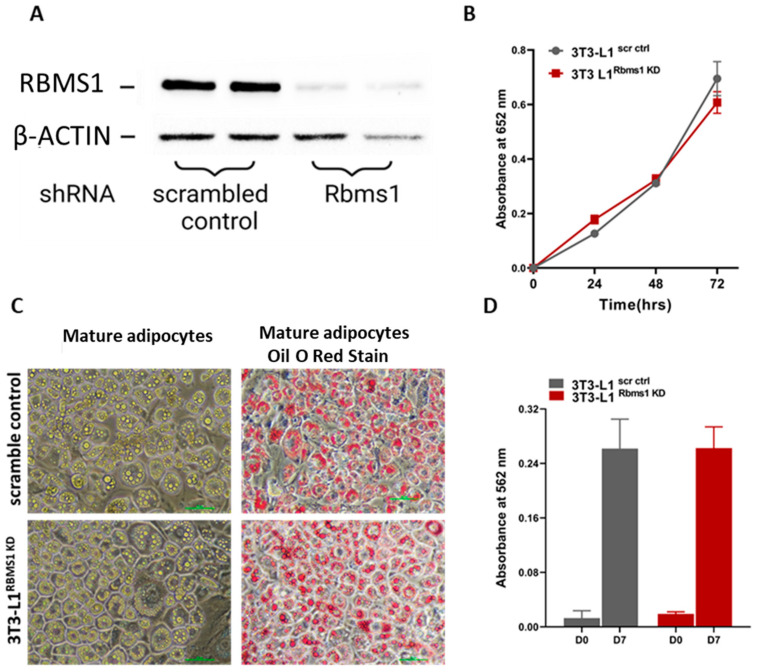
Knockdown of RBMS1 does not alter cellular proliferation or adipogenic differentiation of 3T3-L1 cells. (**A**) A representative blot showing the expression of RBMS1 in cells stably expressing either scrambled shRNA or *Rbms1* shRNA. (**B**) The proliferation and viability of scr ctrl and RBMS1KD 3T3-L1 pre-adipocytes as measured using MTT assay. (**C**) Representative images showing scr ctrl and RBMS1KD 3T3-L1 adipocytes before and after oil red O staining. (**D**) Quantification of lipid content of scr ctrl and RBMS1KD 3T3-L1 cells before (day 0) and after adipogenic differentiation (day 7). Each experiment is a representative of at least three independent experiments.

**Figure 4:**
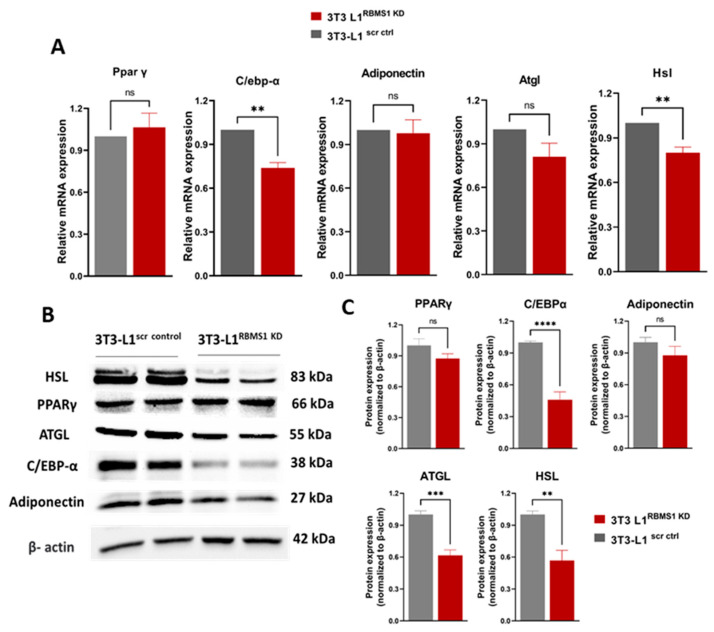
Knockdown of RBMS1 impacts the expression of key adipogenic markers. (**A**) Bar graphs showing the mRNA expression of key adipogenic markers in scr ctrl and RBMS1KD 3T3-L1 adipocytes. (**B**) Immunoblot showing the expression of key adipogenic markers and (**C**) the quantification of the protein expression in scr ctrl and RBMS1KD 3T3-L1 adipocytes. Each bar represents the average of at least three independent experiments. Statistical analysis uses the unpaired *t*-test. **** *p* < 0.0001, *** *p* < 0.001, ** *p* < 0.01, and ^ns^ *p* > 0.05.

Given the significant expression of RBMS1 before and during the early stages of the adipogenic differentiation of 3T3 L1 cells, we hypothesized that RBMS1 might play a role in the differentiation process. To test this hypothesis, we used shRNA to silence Rbms1 expression in 3T3-L1 cells using a lentiviral transduction system followed by puromycin selection. RBMS1 knockdown (RBMS1KD) was confirmed by immunoblotting with specific antibodies (Figure 3A). Interestingly, RBMS1 knockdown had no effect on the proliferation and viability of 3T3-L1 cells, as measured using the MTT assay (Figure 3B). Moreover, cells appeared to differentiate into adipocytes with the formation of lipid droplets similar to scrambled control cells (scr ctrl) (Figure 3C). The quantification of oil droplets revealed no difference in the lipid content of RBMS1 knockdown cells compared to the scrambled control (Figure 3D). Furthermore, the mRNA and protein expression of two important markers of adipogenesis, proliferator-activated receptor gamma (PPARγ) and adiponectin, were unaffected by RBMS1 knockdown (Figure 4A–C). However, several other adipogenic markers such as adipose triglyceride lipase (ATGL), CCAAT/enhancer-binding protein alpha (C/EBPα) and hormone-sensitive lipase (HSL) were significantly downregulated in RBMS1 knockdown 3T3-L1 cells compared to the scrambled control (Figure 4A–C). C/EBP α is a master transcription factor that is known to regulate gene expression and plays a crucial role in adipogenic differentiation and development. ATGL and HSL are two key enzymes involved in the breakdown of triglycerides into free fatty acids. They play a crucial role in regulating lipid metabolism and energy homeostasis in the body. ATGL is responsible for the initial step of triglyceride hydrolysis, while HSL acts in the second step and is also involved in the breakdown of other lipid molecules. Interestingly, ATGL expression in RBMS1KD cells is only affected at the protein level, implying that RBMS1 may be involved in the post-transcriptional regulation of ATGL and, thus, regulates lipid mobilization and metabolism.

### 2.4. RNA Sequencing and Downstream Analysis Reveals the Role of RBMS1 in Adipocyte Metabolism

**Figure 5:**
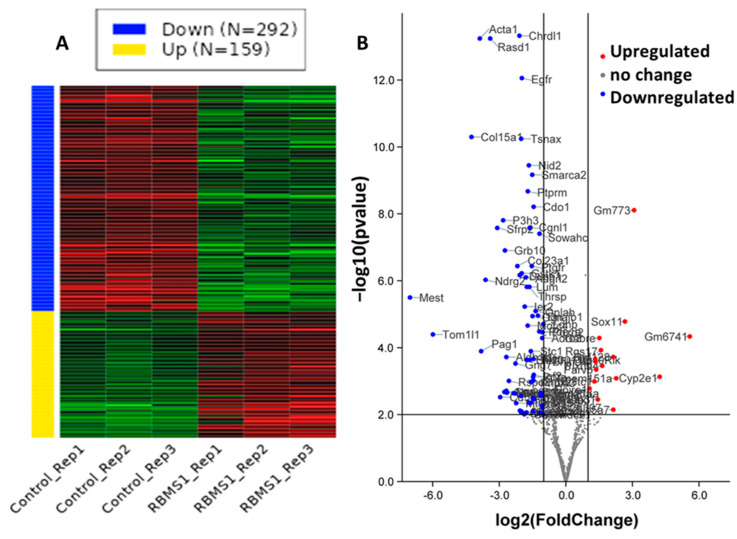
Knockdown of RBMS1 impacts the expression of many genes that are involved in key metabolic functions. (**A**) Heat map showing the differentially expressed genes in the scrambled control and RBMS1KD adipocytes. (**B**) Volcano plot showing differentially expressed genes (FDR adjusted *p*-value < 0.01) in color, with FDR plotted against fold change in adipocytes (2.0). Genes shown in red are upregulated, whereas those in blue are downregulated. Each data point represents data from three biological replicates for each group.

To investigate the effect of RBMS1 deletion on adipocyte gene expression, mature 3T3-L1 cells expressing scrambled control shRNA or *Rbms1* shRNA were subjected to RNA sequencing on the Illumina NovaSeq 6000 platform. DESeq2 and the R package were used to obtain the mRNA expression profile for these two groups. A comparison using a cut-off value of FDR-adjusted *p*-value ≤ 0.1 revealed that 430 genes were differentially expressed in RBMS1 knockdown adipocytes as compared to the scrambled control. The majority of the differentially expressed genes were downregulated (66%), and only 34% were upregulated in RBMS1KD cells compared to the control. Below is a heat map displaying the relative expression levels of the differentially expressed genes across different samples in the two groups (Figure 5A), and the relationship between statistical significance and fold change for each gene is shown in a volcano plot (Figure 5B). The top 25 upregulated and top 25 downregulated genes are shown in Supplementary Tables S2 and S3.

**Figure 6:**
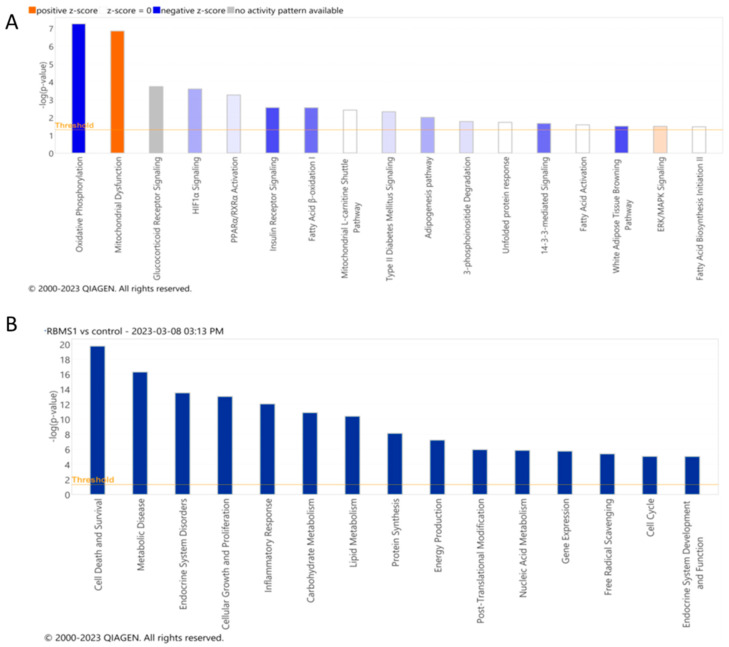
Pathway and biological functions affected by RBMS1 knockdown in adipocytes. (**A**) The most significant signaling and metabolic pathways and (**B**) biological functions identified through IPA analysis of differentially expressed genes in RBMS1KD adipocytes. The ranking is based on the negative log of the ρ value of the enrichment score (upper x–axis) as calculated using IPA. The yellow straight line shows the designated significant threshold −log *p* value = 1.301 (ρ < 0.05) for pathway analysis and −log *p* value = 2 (ρ < 0.01) for biological functions.

The downstream analysis was performed using the Ingenuity Pathway Analysis (IPA) program to identify the biological pathways and networks that are enriched in 3T3 L1 adipocytes after RBMS1 knockdown. The analysis revealed that the differentially expressed genes were primarily involved in pathways such as oxidative phosphorylation, mitochondrial dysfunction, glucocorticoid receptor signaling, hypoxia-inducible factor 1alpha (HIF1) signaling, AMP-activated protein kinase (AMPK) signaling, insulin receptor signaling, fatty acid oxidation, and many others (Figure 6A). Additional functional analysis revealed that RBMS1 knockdown altered cellular activities such as lipid metabolism, glucose metabolism, post-translational modification, cell death and survival, cell growth and proliferation, the inflammatory response, protein synthesis, and energy production (Figure 6B). Our findings imply that RBMS1 regulates a variety of cellular processes in 3T3 L1 adipocytes, including energy metabolism and inflammation.

### 2.5. Differentially Expressed Proteins Associated with RBMS1 Knockdown in Mature Adipocytes

To identify differentially expressed proteins as a result of RBMS1 depletion, samples were subjected to 2D-DIGE proteomic analysis..Three biological replicates from each group (RBMS1KD and scrambled control) were separated, analyzed, and used for the comparative analysis. A representative protein separation pattern is shown in Supplementary Figure S1. A total of 1206 protein spots were analyzed on the gel, and after applying a cut-off line using a *p*-value ≤ 0.05 and a fold change ≥ 1.5, 73 significantly different proteins were identified (Supplementary Figure S2). Subsequently, only 37 proteins could be sequenced and identified using the MALDI-TOF sequencing system combined with the SWISS-PROT database and Mascot software 2.6.00 (Supplementary Table S4). Many proteins had their expression altered only at the protein level, not at the mRNA level, indicating potential post-transcriptional regulation by RBMS1. A comparative analysis of these proteins and their mRNA expression is shown in Figure S3.

**Figure 7:**
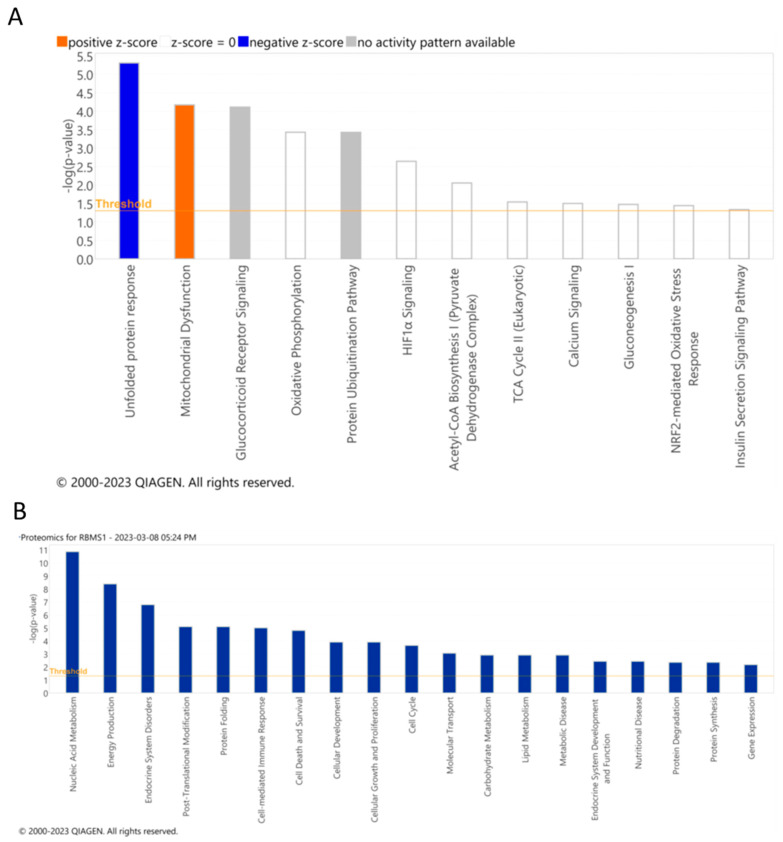
Pathways and biological functions affected identified after analyzing proteomics data from RBMS1KD adipocytes. (**A**) The most significant signaling and metabolic pathways and (**B**) biological functions identified through IPA analysis of differentially expressed proteins in RBMS1KD adipocytes. The ranking is based on the negative log of the ρ value of the enrichment score (upper x–axis) as calculated using IPA. The yellow straight line shows the designated significant threshold −log *p* value = 1.301 (ρ < 0.05).

The list of differentially expressed proteins was uploaded into the IPA database for downstream analysis. The top pathways affected by RBMS1 knockdown included unfolded protein response, mitochondrial dysfunction, and oxidative phosphorylation, consistent with the analysis of the RNA sequencing data (Figure 7A). Additional functional analysis revealed that differentially expressed proteins were significantly enriched in nucleic acid metabolism, energy production, endocrine system disorders, cell cycle, cell death, and lipid metabolism (Figure 7B).

## 3. Discussion

RBPs have emerged as important regulators of adipose metabolism and possible therapeutic targets for metabolic diseases [37,38,39]. These proteins have a distinct structure that allows them to directly bind to RNA and regulate its degradation, stability, adenylation, transport, and, ultimately, protein expression [40]. In the last few years, several members of the RBP family, including HuR, QK, and YBX1, have been shown to regulate adipogenesis by controlling the stability and translation of key mRNAs involved in this process [21,22,23]. RBMS1 is a member of the RNA-binding protein family and contains two conserved RNA binding motifs, RNP1 and RNP2. RBMS1 has been extensively studied for its role in cancer and found to regulate various cellular processes such as alternative splicing, mRNA stability, and translation, making it a potential therapeutic target for cancer treatment [28,29]. Additionally, RBMS1 expression levels have been shown to correlate with cancer progression and patient survival in certain types of cancer. Multiple studies have found that single nucleotide polymorphisms (SNPs) in RBMS1 are linked to the risk of type 2 diabetes mellitus and other obesity-related traits [37,38,41]. Moreover, *Rbms1* mRNA expression shows a significant increase in the placenta from obese mothers as compared to lean mothers [42]. These observations strongly suggest a possible role of RBMS1 in metabolic regulation.

To the best of our knowledge, no prior research has looked into the role of RBMS1 in adipogenesis or adipose tissue metabolism. Therefore, the current study was initiated to investigate the potential role of RBMS1 in adipocyte differentiation and metabolism. We found that RBMS1 is highly expressed in the early stages of differentiation of the murine adipocyte cell line 3T3-L1 and decreases in the later stages. Furthermore, *Rbms1* expression is significantly higher in the subcutaneous and visceral adipose tissue depots of HFD-fed mice as compared to CD-fed mice. Diet-induced obesity (DIO) is known to affect the cellular composition of adipose tissue, which may have a direct influence on the expression of genes such as *Rbms1*. To demonstrate that the increased *Rbms1* expression in response to DIO is not attributable to changes in adipose tissue cellular composition, we re-analyzed an RNA sequencing dataset containing data from adipocytes extracted from visceral adipose tissue of HFD- and CD-fed mice. Consistent with our results from whole adipose tissue, *Rbms1* expression was significantly upregulated in the adipocyte fraction of adipose tissue in response to a high-fat diet. This suggests that the upregulation of *Rbms1* expression in response to a high-fat diet is not due to changes in adipose tissue cellular composition, but rather a direct response to DIO. We further showed that RBMS1 may play a crucial role in regulating the differentiation of 3T3-L1 adipocytes. The depletion of RBMS1 using specific shRNA resulted in the downregulation of some of the key adipogenic markers, including ATGL and HSL, two important lipase enzymes that regulate lipid mobilization and metabolism. Intriguingly, ATGL expression in RBMS1KD cells was only altered at the protein level, not at the mRNA level, suggesting that RBMS1 may control ATGL post-transcriptionally. ATGL is a key enzyme in the conversion of stored fat into energy, and any disruption in its regulation can result in metabolic disorders such as obesity and diabetes [43,44]. More research is needed to determine whether RBMS1 regulates ATGL translation and, if so, how. The transcriptomic and proteomic analyses further revealed that the depletion of RBMS1 led to altered expression of genes involved in key metabolic pathways, including those involved in carbohydrate and lipid metabolism. Interestingly, the mRNA expression of several differentially expressed proteins was unaffected by RBMS1 knockdown, suggesting a possible post-transcriptional regulation by RBMS1. More studies are needed to confirm these findings and to understand the mechanism by which RBMS1 controls the translation of these mRNAs.

Taken together, these findings suggest that RBMS1 may play an important role in adipose metabolism by regulating multiple metabolic pathways. More research is needed to fully understand how RBMS1 regulates adipose metabolism and the adipogenesis process. First, these findings must be replicated in animal models, preferably by creating adipose-specific RBMS1 knockout mice. Second, identifying potential RBMS1 downstream target mRNAs could provide valuable insight into the molecular mechanisms underlying its metabolic effects. Finally, identifying specific compounds capable of modulating RBMS1 expression or activity could be a promising avenue for drug development.

## 4. Materials and Methods

### 4.1. Cell Culture and Differentiation Induction

The 3T3-L1 mouse embryo fibroblast pre-adipocytes were purchased from the American Type Culture Collection (ATCC), (Manassas, VA, USA). Cells were maintained and differentiated in Dulbecco’s modified Eagle’s medium (Sigma, St. Louis, MO, USA) supplemented with 10% heat-inactivated fetal bovine serum (Thermo Fisher Scientific, Waltham, MA, USA) and 10,000 U/mL penicillin-streptomycin (Thermo Fisher Scientific, Waltham, MA, USA), and incubated at 37 °C and 5% CO_2_ as described previously. Briefly, cells were grown until confluency then starved for 2–3 days before initiating the differentiation process. Differentiation was induced by incubating cells with the complete medium (DMEM + 10% FBS) containing the adipogenic cocktail (1 μM insulin, 0.25 µM dexamethasone, 0.5 mM IBMX, 3 µM Rosiglitazone). The differentiation medium was replenished after 3 days with complete medium +1 µM insulin for 24 h. Differentiated cells were maintained in complete medium and used on day 7, as described before [45].

### 4.2. Animals and Diet

C57Bl/6J male mice were a kind gift from Dr. Jahangir Iqbal of the King Abdullah International Medical Research Center in AL-hasaa, SA. Mice were maintained and housed at a 22 ± 0.5 °C temperature, as mentioned previously [46]. Mice were fed ad libitum either a chow diet CD (10% energy by fat, catalog #D12450B, Research Diets, Inc., New Brunswick, NJ, USA) or a high-fat diet HFD (60% energy by fat, catalog #D12492, Research Diets, Inc., New Brunswick, NJ, USA). Four mice in each group were fed the indicated diets for 18 weeks. Weekly weight measurements were recorded, and blood triglycerides, glucose, and free fatty acids were measured. At the end of the treatment, mice were euthanized, and all fat pads were removed and weighed. Experiments were managed with the approval of the KAIMRC Institutional Animal Care and Use Committee (Protocol #s RA17-013-A and RA20-005-A).

### 4.3. Tissue Digestion, RNA Isolation, and RT-PCR

An RNeasy mini kit was purchased from Qiagen, (Germantown, MD, USA) and utilized to isolate RNA from cell lysates. Pre-adipocytes and mature adipocytes were collected, and total RNA was extracted following the manufacturer’s protocol. Meanwhile, RNA from fat tissue was extracted by utilizing Trizole-chloroform following the manufacturer’s protocol, as described previously. In short, an equal amount of total RNA was used to synthesis cDNA using a High-Capacity cDNA Reverse Transcription Kit (Applied Biosystems, Waltham, MA, USA) and was mixed with Power SYBR Green PCR Master Mix (Applied Biosystems, Foster City, CA, USA). The amplification reaction was performed in an Applied Biosystems QuantStudio (TM) 6 Flex real-time PCR System. Primers are listed in the Supplementary Materials (Table S6).

### 4.4. Plasmid, Lentivirus Preparation, and Generation of Stable RBMS1 Knockdown

3T3-L1 knockdown cells were generated using lentiviral transduction and *Rbms1*-specific short hairpin RNA (shRNA). Lentiviral particles were packaged in human embryonic kidney 293T (HEK293T) cells following transfection with the relevant shRNA and lentiviral packaging mix. 3T3-L1 cells were plated in 6-well plates in full medium at 40–50% confluency, and lentiviral particles with transduction enhancer (Polybrene 8 µg/mL) were added to each well. The medium containing viral particles was removed after 48 h of incubation and replaced with fresh medium containing 2 µg/mL puromycin to generate stable RBMS1KD in 3T3-L1 cells.

### 4.5. Library Preparation and RNA Sequencing

RNA was extracted from freshly collected cell pellets as described above. Only samples with a high-quality RNA integrity (RIN ≥ 9) on an Agilent 2100 bioanalyzer (Agilent Technologies, Santa Clara, CA, USA) were judged to be suitable for RNA library preparation prior to whole-transcriptome sequencing. Three biological replicates from each group were utilized for RNA sequencing at the King Abdullah International Medical Research Center’s medical genomics laboratory (Riyadh, Saudi Arabia). In short, the library was created using the Illumina Stranded Total RNA Prep protocol. After obtaining purified total RNA at a concentration of 1000 ng per sample, ribosomal RNA (rRNA) was enzymatically depleted using an Illumina Ribo-zero plus rRNA depletion kit, followed by fragmentation and random hexanucleotide treatment to prime the sample for cDNA synthesis. RNA fragments primed with hexamer were then used to create double-stranded cDNA. To prevent them from ligating each other, adenylate 3’ ends were added, and a ligate anchor was added to prepare a cDNA fragment for dual indexing primers (i7, i5) using RNA prep ligation, IDT DNA/RNA UD indexes, and RNA index anchor kits (New England BioLabs, Ipswich, MA, USA). The library was amplified using PCR and cleaned with Agencourt AMPureXP (Beckman, CA, USA). The final library was then normalized, pooled, and loaded into an SP flow cell for up to 400 million reads of coverage. On an Illumina NovaSeq 6000 platform, the library was sequenced (200 cycles).

### 4.6. RNA Sequencing Data Processing

The generated raw sequence reads were trimmed using Trim Galore (version 0.6.7) [47] to remove adapter sequences and low-quality bases. Quality assessment was performed using FastQC (version 0.11.9) to evaluate the overall quality of the reads, including measures such as per-base sequence quality scores, sequence length distribution, and GC content. Processed reads were aligned to the Ensembl reference genome GRCm38 using STAR (version 2.7.10a) [48], a spliced transcripts aligner, with default parameters. Gene-level counts were generated from the aligned reads using Salmon (version 1.9.0) [49], a fast and accurate transcript quantification tool, with default parameters. Differentially expressed genes (DEGs) between the study groups were identified using DESeq2 (version 1.38.0) [50], an R package for RNA-seq data analysis. DESeq2 was used to calculate the fold change, *p*-value, and false discovery rate (FDR) for each gene, with the threshold for significance set at an FDR-adjusted *p*-value < 0.05. Batch effects were investigated using principal component analysis (PCA). Logarithmic scale (base 2) fold change was used to determine the magnitude of gene expression changes. DEG lists for each group were uploaded to Ingenuity Pathways Analysis (IPA), a bioinformatics tool for functional mapping and pathway analysis. IPA was used to identify enriched biological functions, pathways, and networks associated with the DEGs, using default settings and a significance threshold of *p*-value < 0.05. All statistical analyses were performed using R (version 4.2.1) unless otherwise stated. Heat maps were generated using the integrated Differential Expression and Pathway analysis (iDEP.96) platform [51]. TBtools 1.098 software was used to extract data of the entire repertoire of RNA-binding proteins and to generate volcano plots from RNA sequencing data [52]. All raw sequencing data has been deposited to Gene Expression Omnibus record number GSE233256.

### 4.7. RNA Sequencing Re-Analysis

The GSE142187 dataset was analyzed using the GEO RNA-seq Experiments Interactive Navigator (GREIN) platform [53]. Dataset GSE142187 contained RNA expression data from adipocytes isolated from the white adipose tissue of mice fed either a regular chow or high-fat diet for 8 and 20 weeks [36]. CPM-normalized mRNA expression was obtained from the GREIN platform, and differential gene expression was estimated. Raw *p* values were adjusted for multiple testing using the Benjamini–Hochberg procedure and only adjusted *p* (Padj) values lower than 0.05 were considered significant.

### 4.8. Cellular Localization through Confocal Microscopy

Pre- and post-differentiated 3T3-L1 cells were plated in glass-bottom dishes, washed with PBS, and fixed with 4% paraformaldehyde. Cells were permeabilized by incubating with PBS containing 0.1% Triton X-100 and incubated in blocking solution (1% BSA, 22.52 mg/mL glycine) in PBST (PBS + 0.1% Tween 20). Cells were then incubated with RBMS1 antibody for 1 h at room temperature followed by washing and incubation with goat anti-rabbit IgG (H+L) highly cross-adsorbed secondary antibody for 1 h in the dark. 40,6-diamidino-2-phenylindole (DAPI, Cell Signaling Technology, Danvers, MA, USA) was used to stain nuclei in cell cultures. The cells were imaged using an Eclipse TE2000 confocal laser scanning microscope (CLSM) (Nikon Corporation, Tokyo, Japan).

### 4.9. Measurement of Cell Growth and Viability

To investigate the impact of the depletion of RBMS1 on the proliferation and viability of 3T3 L1 cells, a 3-(4,5- dimethylthiazol-2-yl)-2,5-diphenyltetrazolium bromide (MTT) assay was performed. Briefly, cells were seeded at a density of 2 × 10^3^ cells/well in 96-well plates and incubated at 37 °C in 5% CO^2^. Then, 5 uL of 0.5 mg/mL MTT (Thermo Fisher Scientific, Waltham, MA, USA) was added to each well after 24, 48, and 72 h of incubation and they were incubated for 4 h in 5% CO_2_ at 37 °C. To dissolve the formazan crystals, 100 uL DMSO (Sigma, St. Louis, MO, USA) was used, and optical density (OD) at 562 nm was measured using a SpectraMax ^®^ Microplate Reader (Molecular Device, San Jose, CA, USA).

### 4.10. Oil Red O Stain and Lipid Quantification

The impact of RBMS1KD on lipid accumulation was measured through oil red O staining and lipid quantification. Briefly, cells were seeded in 12-well plates and were differentiated after they reached confluency, as described before [45]. On day 7, media were removed, and cells were washed and fixed in 4% paraformaldehyde methanol free for 1 h. A freshly made oil red O stain was filtered through a 0.2 µm filter and used to stain cells for 20 min at room temperature. Cells were rinsed with 60% isopropanol for 5 min to remove any unwanted background. The cells were imaged on a phase contrast microscope. To quantify the lipid droplets, 100% isopropanol was used to extract the stain from the droplets, and absorbance was measured at 562 nm using a SpectraMax ^®^ Microplate Reader (Molecular Device, San Jose, CA, USA).

### 4.11. Western Blotting

Proteins from cell pellets were extracted using RIPA buffer (Thermo Fisher Scientific, Waltham, MA, USA) supplemented with a protease inhibitor cocktail (Thermo Fisher Scientific, Waltham, MA, USA). Then, 15 μg protein was separated through 10% sodium dodecyl sulfate polyacrylamide gel electrophoresis (SDS-PAGE) and transferred to a nitrocellulose membrane (0.2 μm, Bio-Rad Laboratories, Philadelphia, PA, USA) using a wet transfer system at 60 V for 1 h at 4 °C. Membranes were blocked using 5% fat-free milk and probed with the indicated primary antibodies, followed by the suitable anti-rabbit/mouse horseradish peroxidase (HRP)-conjugated secondary antibodies. Protein bands were developed and visualized using a ChemiDoc XRS imager (Bio-Rad) with Image Lab software (version 4.1, Bio-Rad).

### 4.12. Protein Extraction and Labelling for Two-Dimensional Electrophoresis and Image Scanning

Three separated replicates from each experimental group were used for proteomics analysis at the Obesity Research Center, College of Medicine at King Saud University (Riyadh, Saudi Arabia). Briefly, mature adipocytes were lysed and sonicated in buffer(1.5 mL, pH 8.8, 30 mM Tris buffer containing 7 M urea, 2 M thiourea, 2% Chaps, 1× protease inhibitor mix), as described previously [54]. In brief, after the sonication, 50 mM dithiothreitol (DTT) was added to stabilize the proteins, followed by 40 min centrifugation at 20,000× *g* at 4 °C. Supernatant was used and then precipitated using a 2D clean-up kit (GE Healthcare, USA) according to the manufacturer’s protocol. A buffer (7 M urea, 2 M thiourea, 4% CHAPS, and 30 mM Tris) was prepared and utilized to re-suspend the protein pellets, followed by protein pH adjustment to 8.5. Protein concentrations were measured in triplicate using a 2D-Quantkit (GE Healthcare, Danderyd, Sweden). After that, proteins were labeled with CyDye™ DIGE Fluor minimal dye (400 pmol/50 µg) (GE Healthcare, Danderyd, Sweden) according to the manufacturer’s recommendation. Each biological replicate was labeled with either cy3 or cy5. Moreover, a pooled standard of 50 µg protein from each of the 6 samples was labeled with cy2. Accordingly, samples were combined and separated by first dimension using Immobiline Dry Strips (24 cm, pH 4–7; GE Healthcare, Danderyd, Sweden). These separated proteins were subsequently separated using second-dimension sodium dodecyl sulfate polyacrylamide gel electrophoresis (SDS-PAGE) (Ettan DALT six vertical units, GE Healthcare, Danderyd, Sweden), scanned with a Sapphire Biomolecular Imager (Azure Bio systems, Dublin, OH, USA), and digitalized via the image analysis software Sapphire Capture system Version 1.12.0921.0 (Azure Biosystems, Dublin, OH, USA). In order to identify the statistically significant protein spots, cy2 labeling (internal standard) was included to enable normalization across gels and quantitative differential analysis of the protein levels. An ANOVA parameter was used to determine the significant changes in protein abundance by applying a cut-off line *p*-value ≤ 0.05 and fold change ≥ 1.5. Subsequently, a total protein of 1 mg was created by pooling equal protein amounts from 6 samples to prepare a preparative gel (3 knockdown, 3 controls). The gel was stained for five days with Coomassie stains (Thermo Fisher Scientific, Waltham, MA, USA), followed by a gentle rinse with distilled water. The stained gel was kept at 4 °C until the protein spots could be selected, picked, and identified using MS.

### 4.13. MALDI-TOF Mass Spectrometry and Data Processing

Statistically significant protein spots were collected manually, washed, and digested according to previously described methods [55,56]. Trypsin was used to cleave protein peptides at 37 °C overnight. Next, 0.8 µL of tryptic peptides was loaded into a MALDI target plate (384 MTP Anchorchip, 800 µm Anchorchip, Bruker Daltonics, Bremen, Germany). MALDI-MS/MS spectra were measured on an UltraflexTerm TOF mass spectrometer connected to a LIFT-MS/MS device (Bruker Daltonics). MS data were translated using BioTools v3.2 (Bruker Daltonics). Each peptide sequence was aligned to the database to identify proteins by utilizing the Mascot engine (v2.0.04, updated on 9 May 2021; Matrix Science Ltd., London, UK). Only those proteins that showed a Mascot score greater than 56 with *p* ≤ 0.05 were considered for Ingenuity Pathway Analysis (IPA).

### 4.14. Pathways Analysis and Functional Ontology

The functional analysis and pathway analysis were performed through the use of IPA (QIAGEN Inc. https://www.qiagenbioinformatics.com/products/ingenuity-pathway-analysis (accessed on 8 March 2023)). Only differentially expressed genes and proteins were uploaded for core analysis. In short, a list of DE genes containing the ratio-transformed log_2_ fold change and adjusted *p*-value for each gene was imported, and core analysis was run with consideration of fold change less than or equal to 1.5 and *p*-value 0.1 and 0.05 for proteomics. Bio-functional analysis was carried out to characterize their functions.

### 4.15. Antibodies and Primers

Primary antibodies against RBMS1 (#150353) were purchased from Abcam (Cambridge, UK). ATGL (Cell Signaling Technology (#2439S), PPARγ (Invitrogen, Waltham, MA, USA (#PA3-821A), C/ebp α (Cell Signaling Technology (#8178S), HSL (Cell Signaling Technology (#4107S), and Adiponectin (Invitrogen #MA1-054)). Details are listed in (Table S5).

### 4.16. Statistical Analysis

Each experiment was carried out three independent times for two experimental groups. A total of at least 3 technical/biological replicates were statistically analyzed using the two-tailed unpaired *t*-test or analysis of variance (ANOVA) using Prism GraphPad 9 software (GraphPad Software, Inc., La Jolla, CA, USA). Only values that showed a *p*-value less than or equal to 0.05 were considered significant.

## 5. Conclusions

In conclusion, the current study identifies RBMS1 as an important regulator of adipogenesis and adipose metabolism. These preliminary findings must be confirmed in animal models, and the use of adipose-specific RBMS1 knockout mice could provide valuable insight into the functional significance of this protein.

## Data Availability

The datasets generated during the current study has been deposited to the Gene Expression Omnibus record number GSE138242.

## Supplementary Materials

The supporting information can be downloaded at: https://www.mdpi.com/article/10.3390/ijms241411300/s1.

## References

[1] Zhang Z.Y., Wang M.W. (2012). Obesity, a health burden of a global nature. Acta Pharmacol. Sin..

[2] Kelly T., Yang W., Chen C.S., Reynolds K., He J. (2008). Global burden of obesity in 2005 and projections to 2030. Int. J. Obes..

[3] Kopelman P.G. (2000). Obesity as a medical problem. Nature.

[4] Conway B., Rene A. (2004). Obesity as a disease: No lightweight matter. Obes. Rev..

[5] Da Costa L.A., Arora P., García-Bailo B., Karmali M., El-Sohemy A., Badawi A. (2012). The association between obesity, cardiometabolic disease biomarkers, and innate immunity-related inflammation in canadian adults. Diabetes Metab. Syndr. Obes..

[6] Ghoorah K., Campbell P., Kent A., Maznyczka A., Kunadian V. (2016). Obesity and cardiovascular outcomes: A review. Eur. Heart J. Acute Cardiovasc. Care.

[7] Guh D.P., Zhang W., Bansback N., Amarsi Z., Birmingham C.L., Anis A.H. (2009). The incidence of co-morbidities related to obesity and overweight: A systematic review and meta-analysis. BMC Public Health.

[8] Wang Z.J., Zhou Y.J., Galper B.Z., Gao F., Yeh R.W., Mauri L. (2015). Association of body mass index with mortality and cardiovascular events for patients with coronary artery disease: A systematic review and meta-analysis. Heart.

[9] Olsen C.M., Green A.C., Whiteman D.C., Sadeghi S., Kolahdooz F., Webb P.M. (2007). Obesity and the risk of epithelial ovarian cancer: A systematic review and meta-analysis. Eur. J. Cancer.

[10] Sun J.W., Zhao L.G., Yang Y., Ma X., Wang Y.Y., Xiang Y.B. (2015). Obesity and risk of bladder cancer: A dose-response meta-analysis of 15 cohort studies. PLoS ONE.

[11] Mohammad S., Aziz R., Al Mahri S., Malik S.S., Haji E., Khan A.H., Khatlani T.S., Bouchama A. (2021). Obesity and COVID-19: What makes obese host so vulnerable?. Immun. Ageing.

[12] Aziz R., Sherwani A.Y., Al Mahri S., Malik S.S., Mohammad S. (2023). Why Are Obese People Predisposed to Severe Disease in Viral Respiratory Infections?. Obesities.

[13] Wang Y., Hou H., Xu J., Wang Y., Yang H. (2022). The association between obesity and ICU admission among COVID-19 patients: A meta-analysis of adjusted risk estimates. Am. J. Emerg. Med..

[14] Longo M., Zatterale F., Naderi J., Parrillo L., Formisano P., Raciti G.A., Beguinot F., Miele C. (2019). Adipose tissue dysfunction as determinant of obesity-associated metabolic complications. Int. J. Mol. Sci..

[15] Van de Woestijne A.P., Monajemi H., Kalkhoven E., Visseren F.L.J. (2011). Adipose tissue dysfunction and hypertriglyceridemia: Mechanisms and management. Obes. Rev..

[16] Kawai T., Autieri M.V., Scalia R. (2021). Adipose tissue inflammation and metabolic dysfunction in obesity. Am. J. Physiol. Cell Physiol..

[17] Unamuno X., Gómez-Ambrosi J., Rodríguez A., Becerril S., Frühbeck G., Catalán V. (2018). Adipokine dysregulation and adipose tissue inflammation in human obesity. Eur. J. Clin. Investig..

[18] Kunz H.E., Hart C.R., Gries K.J., Parvizi M., Laurenti M., Man C.D., Moore N., Zhang X., Ryan Z., Polley E.C. (2021). Adipose tissue macrophage populations and inflammation are associated with systemic inflammation and insulin resistance in obesity. Am. J. Physiol. Endocrinol. Metab..

[19] Luo L., Liu M. (2016). Adipose tissue in control of metabolism. J. Endocrinol..

[20] Sarjeant K., Stephens J.M. (2012). Adipogenesis. Cold Spring Harb. Perspect. Biol..

[21] Ahmad B., Serpell C.J., Fong I.L., Wong E.H. (2020). Molecular Mechanisms of Adipogenesis: The Anti-adipogenic Role of AMP-Activated Protein Kinase. Front. Mol. Biosci..

[22] De sá P.M., Richard A.J., Hang H., Stephens J.M. (2017). Transcriptional regulation of adipogenesis. Compr. Physiol..

[23] Rosen E.D., MacDougald O.A. (2006). Adipocyte differentiation from the inside out. Nat. Rev. Mol. Cell Biol..

[24] Squillaro T., Peluso G., Galderisi U., Di Bernardo G. (2020). Long non-coding RNAs in regulation of adipogenesis and adipose tissue function. eLife.

[25] Maeda R., Kami D., Shikuma A., Suzuki Y., Taya T., Matoba S., Gojo S. (2021). RNA decay in processing bodies is indispensable for adipogenesis. Cell Death Dis..

[26] Wei S., Du M., Jiang Z., Hausman G.J., Zhang L., Dodson M.V. (2016). Long noncoding RNAs in regulating adipogenesis: New RNAs shed lights on obesity. Cell. Mol. Life Sci..

[27] Zhang P., Wu W., Ma C., Du C., Huang Y., Xu H., Li C., Cheng X., Hao R., Xu Y. (2022). RNA-Binding Proteins in the Regulation of Adipogenesis and Adipose Function. Cells.

[28] Siang D.T.C., Lim Y.C., Kyaw A.M.M., Win K.N., Chia S.Y., Degirmenci U., Hu X., Tan B.C., Walet A.C.E., Sun L. (2020). The RNA-binding protein HuR is a negative regulator in adipogenesis. Nat. Commun..

[29] Wu R., Cao S., Li F., Feng S., Shu G., Wang L., Gao P., Zhu X., Zhu C., Wang S. (2022). RNA-binding protein YBX1 promotes brown adipogenesis and thermogenesis via PINK1/PRKN-mediated mitophagy. FASEB J..

[30] Lu H., Ye Z., Zhai Y., Wang L., Liu Y., Wang J., Zhang W., Luo W., Lu Z., Chen J. (2020). QKI regulates adipose tissue metabolism by acting as a brake on thermogenesis and promoting obesity. EMBO Rep..

[31] Zhang W., Sun Y., Bai L., Zhi L., Yang Y., Zhao Q., Chen C., Qi Y., Gao W., He W. (2021). RBMS1 regulates lung cancer ferroptosis through translational control of SLC7A11. J. Clin. Investig..

[32] Zhang J., Zhang G., Zhang W., Bai L., Wang L., Li T., Yan L., Xu Y., Chen D., Gao W. (2022). Loss of RBMS1 promotes anti-tumor immunity through enabling PD-L1 checkpoint blockade in triple-negative breast cancer. Cell Death Differ..

[33] Dankert J.T., Wiesehöfer M., Wach S., Czyrnik E.D., Wennemuth G. (2020). Loss of RBMS1 as a regulatory target of miR-106b influences cell growth, gap closing and colony forming in prostate carcinoma. Sci. Rep..

[34] Carter H. (2020). Loss of rna-binding protein rbms1 promotes a metastatic transcriptional program in colorectal cancer. Cancer Discov..

[35] Jin L., Zhang Y., Jiang Y., Tan M., Liu C. (2022). Circular RNA Rbms1 inhibited the development of myocardial ischemia reperfusion injury by regulating miR-92a/BCL2L11 signaling pathway. Bioengineered.

[36] Jones J.E.C., Rabhi N., Orofino J., Gamini R., Perissi V., Vernochet C., Farmer S.R. (2020). The Adipocyte Acquires a Fibroblast-Like Transcriptional Signature in Response to a High Fat Diet. Sci. Rep..

[37] Silva A.N.A., Nicchio I.G., da Silva B.R., Martelli M.G.G., Hidalgo M.A.R., Nepomuceno R., Theodoro L.H., Cirelli J.A., Orrico S.R.P., Cirelli T. (2022). Polymorphisms in risk genes of type 2 diabetes mellitus could be also markers of susceptibility to periodontitis. Arch. Oral Biol..

[38] Qi L., Cornelis M.C., Kraft P., Stanya K.J., Kao W.H.L., Pankow J.S., Dupuis J., Florez J.C., Fox C.S., Paré G. (2010). Genetic variants at 2q24 are associated with susceptibility to type 2 diabetes. Hum. Mol. Genet..

[39] LLeonart M.E. (2022). Understanding RNA-binding proteins. Semin. Cancer Biol..

[40] Wurth L., Gebauer F. (2015). RNA-binding proteins multifaceted translational regulators in cancer. Biochim. Biophys. Acta Gene Regul. Mech..

[41] Kazakova E.V., Chen M., Jamaspishvili E., Lin Z., Yu J., Sun L., Qiao H. (2018). Association between RBMS1 gene rs7593730 and BCAR1 gene rs7202877 and Type 2 diabetes mellitus in the Chinese Han population. Acta Biochim. Pol..

[42] Alvine T., Dhasarathy A., Bundy A., Bhattacharya A., Darland D., Hur J., Perley D., Johnson L., Rusten M., Roemmich J. (2019). RBMS1 Methylation and mRNA Expression Are Differentially Regulated in Placenta Tissue from Obese Women (P11-131-19). Curr. Dev. Nutr..

[43] Schreiber R., Xie H., Schweiger M. (2019). Of mice and men: The physiological role of adipose triglyceride lipase (ATGL). Biochim. Biophys. Acta Mol. Cell Biol. Lipids.

[44] Smirnova E., Goldberg E.B., Makarova K.S., Lin L., Brown W.J., Jackson C.L. (2006). ATGL has a key role in lipid droplet/adiposome degradation in mammalian cells. EMBO Rep..

[45] Al Mahri S., Okla M., Rashid M., Malik S.S., Iqbal J., Al Ibrahim M., Dairi G., Mahmood A., Muthurangan M., Yaqinuddin A. (2023). Profiling of G-Protein Coupled Receptors in Adipose Tissue and Differentiating Adipocytes Offers a Translational Resource for Obesity/Metabolic Research. Cells.

[46] Otaibi A.A., Mubarak S.A., Qarni A.A., Hawwari A., Bakillah A., Iqbal J. (2022). ATP-Binding Cassette Protein ABCC10 Deficiency Prevents Diet-Induced Obesity but Not Atherosclerosis in Mice. Int. J. Mol. Sci..

[47] Krueger F., James F., Ewels P., Afyounian E. S-BB 201. TrimGalore. https://github.com/FelixKrueger/TrimGalore.

[48] Dobin A., Davis C.A., Schlesinger F., Drenkow J., Zaleski C., Jha S., Batut P., Chaisson M., Gingeras T.R. (2013). STAR: Ultrafast universal RNA-seq aligner. Bioinformatics.

[49] Patro R., Duggal G., Love M.I., Irizarry R.A., Kingsford C. (2017). Salmon provides fast and bias-aware quantification of transcript expression. Nat. Methods.

[50] Love M.I., Huber W., Anders S. (2014). Moderated estimation of fold change and dispersion for RNA-seq data with DESeq2. Genome Biol..

[51] Ge S.X., Son E.W., Yao R. (2018). iDEP: An integrated web application for differential expression and pathway analysis of RNA-Seq data. BMC Bioinform..

[52] Chen C., Chen H., Zhang Y., Thomas H.R., Frank M.H., He Y., Xia R. (2020). TBtools: An integrative toolkit developed for interactive analyses of big biological data. Mol. Plant.

[53] Mahi N.A., Najafabadi M.F., Pilarczyk M., Kouril M., Medvedovic M. (2019). GREIN: An Interactive Web Platform for Re-analyzing GEO RNA-seq Data. Sci. Rep..

[54] Akkour K., Alanazi I.O., Alfadda A.A., Alhalal H., Masood A., Musambil M., Abdel Rahman A.M., Alwehaibi M.A., Arafah M., Bassi A. (2022). Tissue-Based Proteomic Profiling in Patients with Hyperplasia and Endometrial Cancer. Cells.

[55] Benabdelkamel H., Masood A., Almidani G.M., Alsadhan A.A., Bassas A.F., Duncan M.W., Alfadda A.A. (2015). Mature adipocyte proteome reveals differentially altered protein abundances between lean, overweight and morbidly obese human subjects. Mol. Cell. Endocrinol..

[56] Alfadda A.A., Masood A., Al-Naami M.Y., Chaurand P., Benabdelkamel H. (2017). A proteomics based approach reveals differential regulation of visceral adipose tissue proteins between metabolically healthy and unhealthy obese patients. Mol. Cells.

