# Supplementary material for: Transcriptomic and Proteomic Analysis Reveals the Potential Role of RBMS1 in Adipogenesis and Adipocyte Metabolism
Source: Int J Mol Sci. 2023 Jul 11;24(14):11300. doi: 10.3390/ijms241411300 (PMC10379198; doi:10.3390/ijms241411300)
Supplement: Supplementary file 1 [file ijms-24-11300-s001.zip › ijms-2376828-supplementary.pdf]

RBP name	Average CPM
Hdlbp	11.03
Pabpc1	11.03
Srrm2	10.87
Zfp36l1	10.66
Hnrnpa2b1	10.42
Hnrnpa3	10.24
Ncl	10.14
Ankrd17	10.02
Fus	9.98
Eif4b	9.91
Hnrnpf	9.87
Csde1	9.81
Zfp36l2	9.76
Zfr	9.71
Pcbp2	9.69
Hnrnpk	9.68
Ybx1	9.65
Srsf5	9.60
Nono	9.42
Hnrnph1	9.27
Hnrnpa1	9.28
Bicc1	9.25
Igf2bp2	9.19
Rbfox2	9.20
Tnrc6b	9.17
Rbm39	9.13
Syncrip	9.12
Zc3hav1	9.09
Sfpq	9.06
Supt6	8.98
Mbnl1	8.94
**Rbms1**	**8.93**
Rbm25	8.89
Larp1	8.87
Ewsr1	8.86
Larp4	8.79
Mbnl2	8.72

Qk	8.69
Hnrnpd	8.66
Ssb	8.61
Hnrnpdl	8.61
Pcbp1	8.60
Matr3	8.60
Parp14	8.51
Hnrnpab	8.56
Zfp638	8.56
Srrm1	8.55
Zc3h7b	8.55
Pum2	8.54
Hnrnpm	8.50
Sf1	8.52
Eif4h	8.51
Rbm3	8.47
Fmr1	8.42
Ptbp1	8.45
Hnrnpl	8.44
Rc3h2	8.41
Rbms2	8.37
Khsrp	8.36
G3bp2	8.35
Rbms3	8.32
Srsf11	8.31
Srsf6	8.27
Tra2b	8.23
Acin1	8.21
Srsf1	8.14
Srsf3	8.10
Sfswap	8.08
Eif3b	8.09
Taf15	8.06
Rbm5	8.08
Zc3h14	8.07
Spen	8.04
Ankhd1	8.03
Ptbp3	8.02
Hnrnpc	8.00
Srrt	8.00
Tia1	8.00

Marf1	7.99
Khdrbs1	7.94
Zc3h15	7.92
Ccar1	7.91
Raly	7.88
Celf1	7.88
Ythdc1	7.86
Tardbp	7.86
Hnrnpr	7.78
Scaf8	7.85
Helz	7.76
Cstf2t	7.74
Rc3h1	7.73
Srsf10	7.72
Pum1	7.71
Tnrc6a	7.70
Snrnp70	7.71
Safb	7.67
Fubp3	7.64
Upf1	7.65
Hnrnpa0	7.61
U2surp	7.62
Zc3h13	7.60
Ybx3	7.61
Ythdf3	7.60
Igf2bp3	7.61
Ppargc1a	7.56
Cnot4	7.48
Ireb2	7.57
Tnrc6c	7.54
Fubp1	7.51
Fxr1	7.50
Rbm17	7.43
Hnrnph2	7.47
Rbm33	7.43
Pum3	7.46
Rbpms	7.46
Rbm28	7.45
Setd1a	7.40
Ppp1r10	7.40
Rbm6	7.39

Cherp	7.36
Larp4b	7.39
Celf2	7.35
Dazap1	7.34
Tra2a	7.30
Puf60	7.33
Rbm26	7.32
G3bp1	7.32
Zc3h18	7.30
Cpsf6	7.29
U2af2	7.30
Ro60	7.28
Poldip3	7.25
Parp12	7.26
Cpsf7	7.26
Safb2	7.19
Rbmxl1	7.22
Pabpc4	7.19
Snrpd3	7.21
Pabpn1	7.19
Tiparp	7.18
Srsf2	7.18
Cpeb4	7.13
Grsf1	7.14
Aco1	7.13
Zfp36	7.11
Ttc14	7.02
Scaf4	7.06
Zc3h4	7.05
Pdcd11	7.02
Rbm27	6.98
Sart3	6.94
Zc3h7a	6.95
Srek1	6.95
Eif2s1	6.94
Ythdf1	6.89
Mex3a	6.87
Mex3c	6.88
Myef2	6.88
Rbm10	6.86
Samd4	6.84

Srsf9	6.82
Raver1	6.84
Tial1	6.80
Ptbp2	6.79
Rbm15	6.77
Elavl1	6.74
Htatsf1	6.59
Dhx57	6.73
Synj1	6.70
Pprc1	6.65
Akap1	6.62
Srsf4	6.62
Hnrnpll	6.58
Slbp	6.63
Zc3h12a	6.59
Zrsr2	6.59
Enox1	6.57
Dhx8	6.55
Ythdf2	6.52
Pnpt1	6.50
Rbm18	6.47
Prpf3	6.48
Sf3a1	6.32
Ppil4	6.45
Cpne1	6.42
Rbm8a	6.41
Snrpc	6.38
Sugp1	6.38
Pcbp4	6.36
Snrpd1	6.17
Larp7	6.31
U2af1	6.34
Sltm	6.29
Snrpb2	6.27
Rbm22	6.31
Upf3b	6.29
Fxr2	6.26
Tmem63a	6.27
Cstf2	6.30
Msi2	6.25
Zcrb1	6.26

Tep1	6.26
Eif3g	6.26
Snrpa	6.17
Alyref	6.17
Mcm3ap	6.18
Rbm7	6.13
Uhmk1	6.11
Rnps1	5.91
Parn	6.11
Pspc1	6.08
Nifk	6.08
Nol8	6.05
Unk	6.02
Thumpd3	5.85
Snrpf	5.99
Mex3d	5.96
Krr1	6.03
Igf2bp1	6.01
Rrp7a	5.95
Sf3b6	5.95
Sf3b4	5.90
Trmt1l ENSMUSG00000053286	5.86
Dus3l	5.83
Srsf7	5.88
Eif2d	5.85
Snrpb	5.85
Slirp	5.83
Prr3	5.57
Zc3h6	5.76
Lsm2	5.75
Srbd1	5.71
Rbm14	5.28
Zgpat	5.66
Rbm45	5.55
Mkrn1	5.65
Rbm4b	5.55
Sugp2	5.46
Dkc1	5.62
Gtf3a	5.60
Zc3hav1l	5.61
Lsm8	5.52

Thumpd1	5.55
Alkbh8	5.55
Rbmx	5.51
Gm7964	5.25
Trmt1	5.46
Lsm3	5.51
Nelfe	5.46
Hnrnph3	5.42
Zrsr1	5.33
Rbm12b2	5.33
Zcchc17	5.33
Rbm42	5.37
Tut1	5.31
Rbm19	5.23
Trmt2a	5.30
Aptx	5.06
Samd14	5.24
Toe1	4.99
Lsm4	5.08
Clasrp	5.17
Carhsp1	5.16
Nip7	5.14
Lsm1	5.11
Zc3h3	5.08
Lsm6	5.05
Rbm41	5.03
Tdrkh	5.01
Zc3h12c	4.96
Cpsf4	4.89
Mkrn2	4.96
Cirbp	4.96
Synj2	4.92
Naa38	4.68
Rbm34	4.88
Mthfsd	4.84
Snrpg	4.80
Trnau1ap	4.79
Rbm43	4.73
Ascc1	4.66
Cpeb2	4.69
Cpeb3	4.51

Dgkq	4.63
Zc3h12b	4.55
Lemd3	4.47
Ppie	4.54
Nufip1	4.50
Polr2g	4.50
Pno1	4.40
Snrnp35	4.32
Mcts1	4.06
Pcbp3	4.34
Nupl2	3.66
Kin	4.06
Snrpd2	4.14
Lsm10	4.14
Rbmx2	4.16
Larp6	4.13
Mcts2	3.83
Ythdc2	3.95
Mex3b	3.73
U2af1l4	3.84
Rnpc3	3.72
Rbm47	3.79
Celf5	3.79
Unkl	3.61
Ppargc1b	3.70
Nop9	3.70
Leng9	3.62
Mbnl3	3.45
Lsm7	2.60
Rexo5	3.30
Enox2	3.30
Thumpd2	3.30
Akap17b	3.19
Zc3h8	2.95
Dnajc17	3.06
Snrpert	2.67
Zc3h10	2.72
Rbm38	2.72
Alyref2	2.72
Gm2531	2.24
Nsun6	2.18

Csdc2	2.18
Lsm11	2.57
Gm38393	2.44
Celf4	2.05
Rbpms2	2.37
Rbm20	2.37
Gm9833	2.01
Msi1	2.01
Rnf113a1	1.90
Rdm1	2.10
Cpeb1	1.70
Pabpc1l	1.70
Gm7846	1.67
Zmat5	1.67
Rbm12b1	1.43
Gm7353	1.00
Rcan2	1.00
Mkrn3	1.00
Ybx2	1.00
Elavl3	1.00
Gm10110	1.00
Zfp239	1.00
Gm11847	1.00
E130201H02Rik	1.00
Gm8991	1.00
Zc3h12d	1.00
Nova2	1.00
Rbfox3	1.00
Dnd1	1.00

Supplemental Table S1- The expression of RNA binding proteins (RBPs) in 3T3-L1 cells before differentiation

	Gene name	log2FoldChange	padj	baseMean
1	Chrdl1	-2.09402	4.76E-24	375.5417
2	Acta1	-3.87819	5.76E-14	49.61433
3	Rasd1	-3.41071	5.76E-14	97.30497
4	Egfr	-1.98342	8.78E-13	290.9481
5	Col15a1	-4.25313	5.04E-11	941.4901
6	Tsnax	-2.01538	5.70E-11	132.1724
7	Nid2	-1.66705	3.56E-10	340.2233
8	Smarca2	-1.51604	6.80E-10	510.8047
9	Ptprm	-1.71434	2.12E-09	209.2896
10	Cdo1	-1.45215	6.16E-09	937.7774
11	P3h3	-2.8276	1.56E-08	49.57788
12	Sfrp2	-3.09291	2.64E-08	61.2085
13	Cgnl1	-1.63825	2.64E-08	366.6287
14	Sowahc	-1.18834	3.93E-08	386.6229
15	Grb10	-2.7464	1.24E-07	57.46836
16	Col23a1	-2.18552	3.64E-07	47.58231
17	Ptgfr	-1.53576	3.64E-07	281.1325
18	Gstk1	-1.98943	5.97E-07	366.6287
19	Dusp1	-2.0816	6.78E-07	418.0667
20	Anxa5	-0.93079	7.49E-07	766.809
21	Adgrl2	-1.80079	7.91E-07	129.6421
22	Ndrg2	-3.62018	9.39E-07	22.24974
23	Lum	-1.76132	1.52E-06	152.2317
24	Thrsp	-1.64427	1.52E-06	1647.547
25	Mest	-7.02723	3.16E-06	16.24745

Supplemental Table S2- Top 25 most significant downregulated genes in 3T3-L1 cells after Rbms1 knockdown

	Gene name	log2FoldChange	padj	baseMean
1	Gm773	3.074423	7.77E-09	61.99956
2	Pcyox1	0.888747	6.78E-07	1752.371
3	Sox11	2.662427	1.65E-05	67.49147
4	Gm6741	5.583851	4.62E-05	17.76817
5	Gabre	1.507848	5.14E-05	144.6738
6	Rgs17	1.580638	0.000119	73.6931
7	Rnf128	2.136338	0.00019	59.97889
8	Pltp	1.321833	0.000217	442.4367
9	Bhlhe41	1.333575	0.00026	112.2864
10	Plxna4	1.636459	0.000348	59.74828
11	Parvb	1.365159	0.000451	94.36023
12	Cyp2e1	4.228098	0.000737	10.99569
13	Tmem151a	2.270955	0.00082	26.64605
14	Sh3tc1	1.283811	0.001015	71.14589
15	Spire1	1.066147	0.001656	98.83157
16	Tmeff1	0.841214	0.002286	141.5828
17	Stbd1	1.440143	0.003533	75.27255
18	Uck2	0.67885	0.004421	234.8702
19	Six4	0.894881	0.005098	171.7154
20	Fmr1	0.705129	0.005247	1187.294
21	Unc5b	0.68615	0.0063	276.9005
22	Xpr1	0.604063	0.006414	474.9455
23	Prelp	0.656263	0.006692	1068.981
24	Slc5a7	2.137867	0.007106	14.0031
25	Bid	0.85912	0.00811	131.879

Supplemental Table S3- Top 25 most significant downregulated genes in 3T3-L1 cells after Rbms1 knockdown

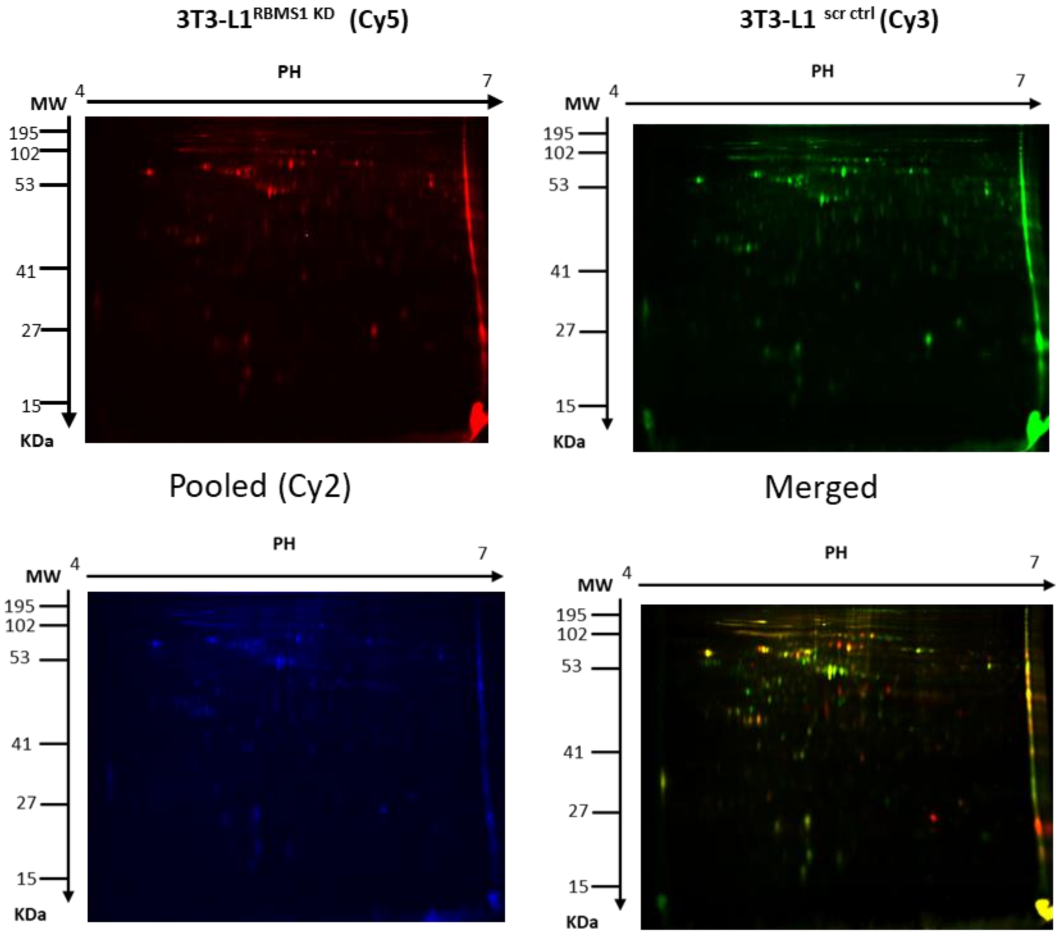

Supplemental Figure S1 (S1)- A representative image showing the protein separation pattern is shown in the 3T3-L1$^{\text{RBMS1 KD and}}$ 3T3-L1 $^{\text{scr ctrl adipocytes.}}$

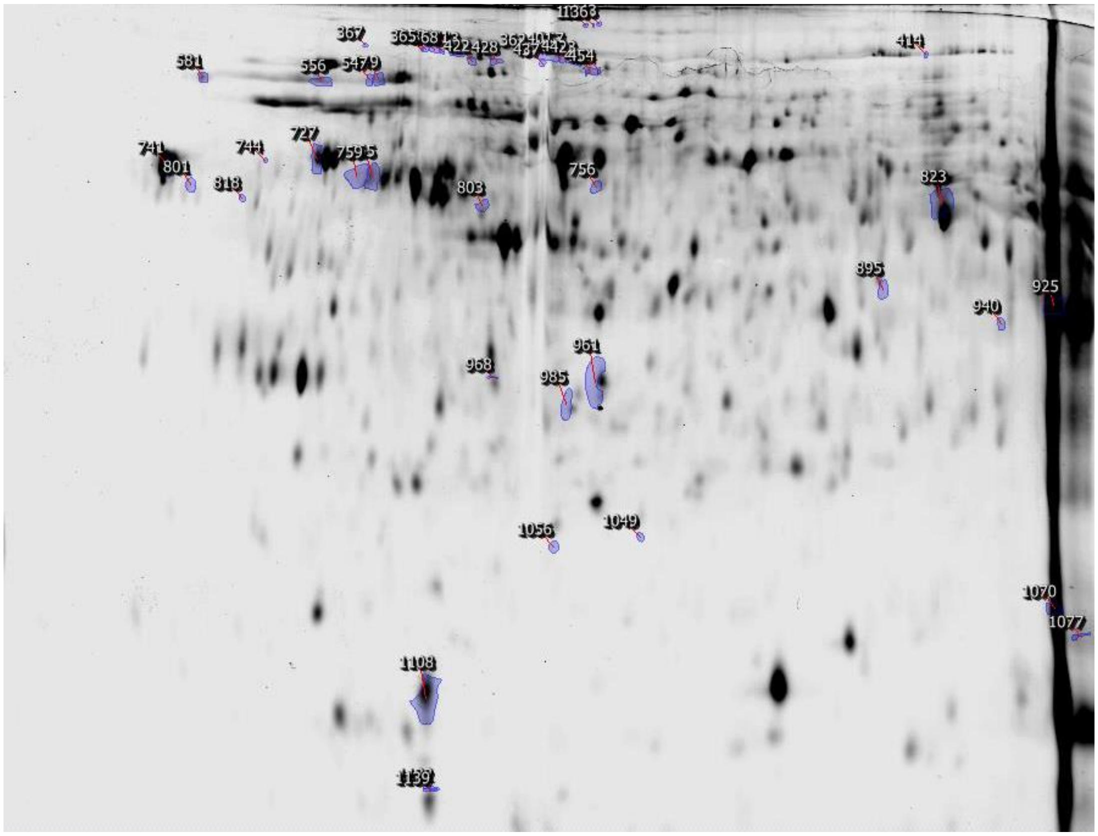

Supplemental Figure S2 (S2)- A representative image protein spots on the gel

Sl no:	Spot No[a]	Accession No[b]	Protein Name	MASCOT ID	P value (ANOVA)	Ratio[c]	Pi[d]	MW[e]	Cov%	Score[f]	EXP
1	1056	Q9DCX2	ATP synthase subunit d, mitochondrial	ATP5H_MOUSE	0.002	-5.04062	5.52	18738	61	57	↓
2	410	P63017	Heat shock cognate 71 kDa protein	HSP7C_MOUSE	0.003	-3.93272	5.37	70827	26	67	↓
3	404	Q8BZQ7	Anaphase-promoting complex subunit 2	ANC2_MOUSE	0.005	-4.76049	5.21	95261	9	57	↓
4	370	P11499	Heat shock protein HSP 90-beta	HS90B_MOUSE	0.005	-4.78179	4.97	83273	39	121	↓
5	744	Q9ET22	Dipeptidyl peptidase 2	DPP2_MOUSE	0.005	-5.30529	5.17	56234	26	200	↓
6	925	P08249	Malate dehydrogenase, mitochondrial	MDHM_MOUSE	0.006	14.29734	8.93	35589	30	58	↑
7	423	P63260	Actin, cytoplasmic 2	ACTG_MOUSE	0.007	-2.93406	5.31	41766	38	57	↓
8	1070	Q6DFW0	Guanine nucleotide exchange factor C9orf72 homolog	CI072_MOUSE	0.007	18.47058	5.4	47284	132	57	↑
9	756	Q3TDX8	Cytochrome b5 reductase 4	NB5R4_MOUSE	0.008	2.964571	6.3	60543	94	80	↑
10	368	P20152	Vimentin	VIME_MOUSE	0.009	-7.76575	5.06	53655	70	347	↓
11	411	P38647	Stress-70 protein, mitochondrial	GRP75_MOUSE	0.01	-2.82905	5.91	73483	44	179	↓
12	895	Q99L13	3-hydroxyisobutyrate dehydrogenase, mitochondrial	3HIDH_MOUSE	0.01	-2.50973	8.37	35417	32	58	↓
13	371	Q6P1G2	Lysine-specific demethylase 2B	KDM2B_MOUSE	0.01	-5.04869	8.66	149638	15	57	↓
14	1132	P07091	Protein S100-A4	S10A4_MOUSE	0.012	-3.89148	5.23	11714	43	70	↓
15	961	P67778	Prohibitin 1	PHB_MOUSE	0.013	-1.98007	5.57	29802	63	99	↓
16	143	Q02566	Myosin-6	MYH6_MOUSE	0.013	4.335798	5.57	223426	36	57	↑
17	362	Q99LD8	N(G),N(G)-dimethylarginine dimethylaminohydrolase 2	DDAH2_MOUSE	0.014	-5.1482	5.66	29627	13	57	↓
18	428	Q91VD9	NADH-ubiquinone oxidoreductase 75 kDa subunit, mitochondrial	NDUS1_MOUSE	0.018	-2.0888	5.51	79698	34	67	↓
19	727	P56480	ATP synthase subunit beta, mitochondrial	ATPB_MOUSE	0.019	-1.81516	5.19	56265	63	165	↓
20	759	Q6P1Y8	Type II inositol 3,4-bisphosphate 4-phosphatase	INP4B_MOUSE	0.02	-2.4315	5.69	100465	20	57	↓
21	369	Q05920	Pyruvate carboxylase, mitochondrial	PYC_MOUSE	0.021	-4.49871	6.25	129602	29	151	↓
22	373	Q6P1Y8	Type II inositol 3,4-bisphosphate 4-phosphatase	INP4B_MOUSE	0.023	-3.874	5.69	100465	15	57	↓
23	1139	Q05920	Pyruvate carboxylase, mitochondrial	PYC_MOUSE	0.026	-3.76159	6.25	129602	22	117	↓
24	365	Q8BY02	NF-kappa-B-repressing factor	NKRF_MOUSE	0.027	-5.21235	9.08	77658	17	57	↓
25	367	Q9JL25	Regulator of G-protein signaling 1	RGS1_MOUSE	0.03	-3.67879	9.2	24091	41	68	↓
26	414	Q8BHX1	HAUS augmin-like complex subunit 1	HAUS1_MOUSE	0.03	-3.92161	5.56	31359	30	62	↓
27	549	Q99LD8	N(G),N(G)-dimethylarginine dimethylaminohydrolase 2	DDAH2_MOUSE	0.037	1.832763	5.66	29627	13	57	↑
28	741	P14211	Calreticulin	CALR_MOUSE	0.039	-2.17188	4.33	47965	27	85	↓
29	765	Q922R8	Protein disulfide-isomerase A6	PDIA6_MOUSE	0.039	-1.8828	5	48070	34	94	↓
30	422	P20152	Vimentin	VIME_MOUSE	0.04	-2.07737	5.06	53655	48	172	↓
31	1108	P16045	Galectin-1	LEG1_MOUSE	0.044	-2.03928	5.32	14856	42	59	↓
32	803	P63260	Actin, cytoplasmic 2	ACTG_MOUSE	0.047	-2.45903	5.31	41766	60	127	↓
33	136	Q32MG2	Protein chibby homolog 2	SPERT_MOUSE	0.048	3.585876	6.28	51833	21	57	↑

34	556	P35486	Pyruvate dehydrogenase E1 component subunit alpha, somatic form, mitochondrial	ODPA_MOUSE	0.049	3.408055	8.49	43204	27	57	↑
35	861	P14206	40S ribosomal protein SA	RSSA_MOUSE	0.05	7.282463	4.8	32817	49	74	↑
36	985	B2RVL6	Zinc finger CCHC domain-containing protein 24	ZCH24_MOUSE	0.051	-1.8505	8.98	26943	33	57	↓
37	1077	Q6P1Y8	Type II inositol 3,4-bisphosphate 4-phosphatase	INP4B_MOUSE	0.058	-4.71116	5.69	104465	15	57	↓

Supplementary Table S4: Differentially abundant proteins between RBMS1 Knockdown vs. Scramble Control . MALDI-TOF mass spectrometry was utilized to identify and analyze the protein spots from the 2D-DIGE gels

[a]Spot numbers matched those included in the 2D-image

[b]Protein accession number for SWISSPROT Database

[c]Protein expression between RBMS1 knockdown vs scramble control (positive values demonstrate an increase in the protein abundance in RBMS1 knockdown; negative values demonstrate a decrease in the protein abundance in RBMS1 knockdown)

[d]Theoretical isoelectric point

[e]Theoretical molecular weight

[f]MASCOT score

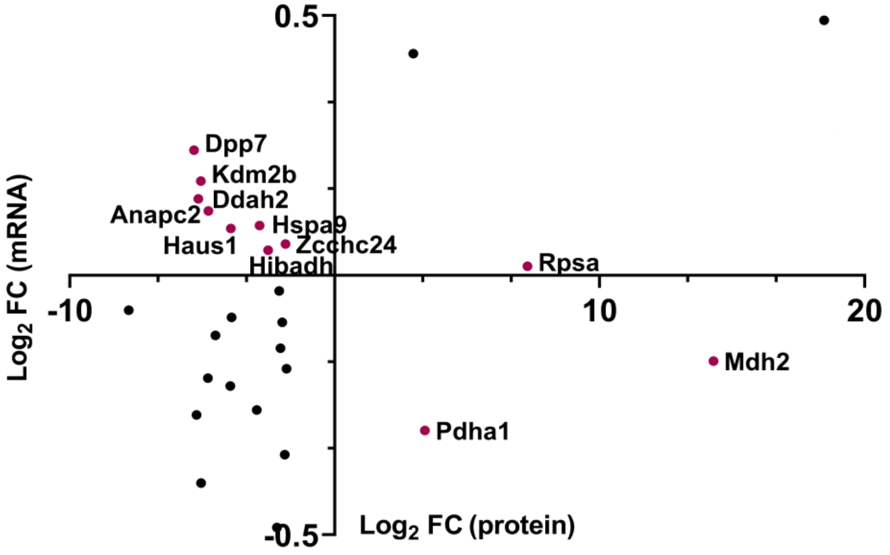

Supplemental Figure S3 (S3). Comparative analysis of these proteins and their mRNA expression (y-axis for log2-fold change in mRNA abundance and x-axis for log2-fold change in protein abundance). Each dot represents a protein/gene and red dots indicate those proteins whose mRNA expression is unchanged or does not align with changes in the protein expression.

Serial	Antibody name	Host/Isotype	Type	Reference
1	β-Actin	Mouse/ IgG2b	Primary Antibody	Invitrogen (MA5-15739)
2	α- Tubulin	Mouse/ IgG1	Primary Antibody	Invitrogen (A11126)
3	GAPDH	Mouse / IgG1	Primary Antibody	Invitrogen **(MA5-15738)**
4	PPARγ	Rabbit/ IgG	Primary Antibody	Invitrogen (PA3-821A)
5	C/EBPα	Rabbit/ IgG	Primary Antibody	Cell Signaling Technology (8178S)
6	ATGL	Rabbit/ IgG	Primary Antibody	Cell Signaling Technology (2439S)
7	Adiponectin	Mouse / IgG	Primary Antibody	Invitrogen (MA1-054)
8	HSL	Rabbit/ IgG	Primary Antibody	Cell Signaling Technology (4107S)
9	Goat Anti-mouse	Goat / IgG	Secondary antibody	Invitrogen (31430)
10	Goat Anti-Rabbi	Goat / IgG	Secondary antibody	Invitrogen (31460)
11	RBMS1	Rabbit/ IgG	Primary Antibody	Abcam(ab150353)

Supplementary Table S5. The list of primary and secondary antibodies used in this study.

Gene Name	Forward (5'-3')	Reverse (5'-3')
Actin	ATGACCCAAGCCGAGAAGG	CGGCCAAGTCTTAGAGTTGTTG
Hsl	GGCAGTGGTGTGTAACTAGGATTG	ATCCATGCTGTGTGAGAACGC
Adipocnectin	AACTTGTGCAGGTTGGATGG	CGATACACATAAGCGGCTT
Atgl	GGAACCAAAGGACCTGATGACC	ACATCAGGCAGCCACTCCAACA
Ppar γ2	GCATGGTGCCTTCGCTGA	TGGCATCTCTGTGTCAACCATG
C/ebp α	CAAGAACAGCAACGAGTACCG	GTCACTGGTCAACTCCAGCAC
RBMS1	CTGAGCAAGACAAACCTCTACAT	GGCCTTATCCAAAATCGCCTT

Supplemental table S6. Sequences of the forward and reverse primers for mouse species ordered from Macrogen and used in the quantitative real time PCR (qRT-PCR*).*
